# Mediating and moderating effects of authoritative parenting styles on adolescent behavioral problems

**DOI:** 10.3389/fpsyg.2024.1336354

**Published:** 2024-02-01

**Authors:** Li Sun, Ao Li, Meng Chen, LuYao Li, Yan Zhao, AnQi Zhu, Peng Hu

**Affiliations:** ^1^College of Education Sciences, Hubei Second Normal College, Wuhan, Hubei, China; ^2^College of Educational Sciences, Hubei Normal University, Huangshi, Hubei, China

**Keywords:** authoritative parenting style, educational anxiety, parent–child relationship, behavioral problems, adolescents

## Abstract

**Introduction:**

This study aimed to analyze how authoritative parenting affects behavioral problems among primary, junior high, and secondary high school students. Today, parental educational anxiety and parent–child relationship conflicts are common in China and are resulting in a high incidence of child behavioral problems. High-quality family education is becoming increasingly important in China. This study sought to provide a reference for developing responsive family education services.

**Methods:**

A total of 10,441 parents in Hubei Province, including urban and rural areas, were evaluated using the Parents’ Education Anxiety Questionnaire, Parental Authority Parenting Questionnaire, Parent–Child Relationship Scale, and Self-Made Behavior Problem Scale to determine the internal mechanisms of child behavioral problems in the family system. To make the sample more representative, this study collected data from primary and secondary schools representative of the southeast, northwest, and center of Hubei Province; further, the number of parents involved in each school was controlled at approximately 300 to ensure that the final sample had analytical value.

**Results:**

Educational anxiety directly affected children’s behavioral problems and indirectly affected them through the conflicts between parent and child. This conflict partially mediated educational anxiety and child behavioral problems, and authoritative parenting played a significant regulatory role in this relationship.

**Discussion:**

Higher levels of educational anxiety among parents increased the likelihood of a depressed family environment. This can lead to deteriorating parent–child relationships, which can result in children’s problem behaviors. Parents can address these problems by changing their approach to education and adjusting their emotions accordingly.

## 1 Introduction

This study explored how parenting styles impact adolescent behavior toward outlining best practices for family education in China. Family education affects child growth and development, family happiness and stability, social harmony and progress, and national prosperity. Family education refers to “social roles (including kinship relations and ancestry), emotions, responsibilities (including child welfare), being together, economics (housekeeping), leisure and care” (Bernardes, 1999; Havigerová et al., 2013). Many countries have introduced laws to promote family education. For example, the U.S. federal government updated and revised the Family Education Protection Act (United Nations, 1989) to emphasize the legal status of family upbringing and provide parents with more educational autonomy.

Meanwhile, the U.K. government published *Guidance for Local Authorities and Parents* (U.K. Government, 1996) to help parents better understand the importance of family upbringing, what parents should focus on, and how they can communicate and work with local authorities. Additionally, the government of Ontario, Canada, issued the *Policy Statement on Family Upbringing* (Ontario Ministry of Education, 2018), which provides a comprehensive overview of family upbringing, clearly expresses the government’s support and endorsement of family upbringing and sets relevant regulatory standards and requirements. The Australian government’s updated *National Education Guidelines* (Australian Government, 2022) outlines numerous requirements and recommendations for family upbringing to regulate its quality and safety effectively. All these documents reflect the international emphasis, concern regarding home education, and provide parents with practical information and legal protection.

Recently, research on family education has emerged along the following lines. First, scholars have considered the impact of family education on child development, particularly the relationships between learning and social, psychological, and physical health. Second, they have explored parents’ roles in family education to uncover how different types of parental involvement impact child development. Third, scholars have investigated heterogeneities in family education; for example, they have studied how families from different ethnic, cultural, and economic backgrounds educate and explore the advantages and disadvantages of different educational approaches. Fourth, scholars have examined parenting skills, examining how to improve parents’ educational skills and knowledge to better support child growth and development. Fifth, researchers have considered views on educational policies, exploring how their attitudes influence their expectations for educational reform (Garcia Yeste et al., 2018).

In China, family education is becoming increasingly important. Historically, family education has played a profound, foundational, and long-term role in the growth of children and youth. Parents’ ability to educate their children affects the effectiveness of family education and child development (Huang Y. et al., 2022). Today, there is a shortage of high-quality family educational strategies, creating contradictions in educational development. Modernization and urbanization have profoundly changed the traditional Chinese family structure (Gao and Bian, 2023). With family structures becoming smaller and more nucleated, elders have less time and energy to devote to family education, and the resources and support they can obtain from within the family are becoming more limited, leading to the absence or devaluation of family education (Qiang, 2021). These trends can cause problems in children and youth. For example, psychological and behavioral issues related to lack of childhood education can exacerbate the trend of under-age delinquency (Danisworo and Wangid, 2022). Some studies have shown that moral and conscious restraints can significantly increase the psychological costs of juvenile involvement in crime. Conversely, low morale and conscious restraints among adolescents increase crime rates; socially maladjusted adolescents are more likely to commit crimes, and associating with transgressive peers can influence adolescent delinquency (Han and Zhang, 2015). Furthermore, emotional problems in adolescents, such as the callousness trait, which manifests itself in insensitivity to negative emotions and a lack of empathy, are key personality factors leading to juvenile delinquency (Ermer et al., 2013).

Further, although providing satisfactory education is a key goal of the new era, parent anxiety around education and parent–child relationship conflicts hinder the widespread implementation of satisfactory family education (Ding and Xue, 2022). Parental educational anxiety is evident in the fact that parents devote all their limited time and energy to their children’s studies. This trend is causing the parent–child relationship to mutate into a teacher–student relationship and reducing the warmth and acceptance characteristics of the original parent–child relationship. This may reduce the child’s sense of security and belonging in their family. Additionally, it may cause children to feel that there is no space for their emotions (Mellon and Moutavelis, 2011). Together, these effects can increase psychological and behavioral problems among children.

Further, the education of both fathers and mothers can directly predict children’s behavioral problems (Gao et al., 2023). Meanwhile, anxiety disorders among parents significantly affect children (Lawrence et al., 2019). Similarly, parental non-pathological anxiety symptoms and traits can also predict emotional problems in children (Bayer et al., 2006). Therefore, it is necessary to reduce parents’ educational anxiety and improve parent–child relationships to improve family education in China. For example, these issues may be addressed by providing parents with accurate educational guidance (Huang et al., 2022a; Zhou Shiyi, 2022), implementing family education legislation, rebuilding family education, and cultivating healthy personalities (Zhang, 2022), thereby alleviating the effects of educational anxiety on children’s behavioral problems and achieving scientific family education.

The ecosystem theory supports the idea that a child’s development is closely related to the education they receive at home. This theory was introduced by Bronfenbrenner (1992), based on his synthesis of the ecological view of development and personal experience. Broadly, the ecosystem theory maintains that the environment greatly influences individual development (Tudge et al., 2009). This theory divides the formative environment into microsystems, mesosystems, exosystems, macrosystems, and ephemeral systems. Ecosystem theory emphasizes the linkages between systems, the bidirectional nature of the role of the individual and the microsystem, the process of the individual’s continuous adjustment to the environment, and the selection and adjustment of the environment; the theory establishes that the environment has a dynamic influence on the individual (Bronfenbrenner, 1992). Within this theoretical framework, family, school, and peers belong to the microsystem. The family system is an environment comprising children, parents, and the family setting, and includes children’s personalities, genders, ages, and parents’ education, occupation, and parenting style (Luan et al., 2013). In the family system, the child directly perceives activities and interacts with their parents. Notably, the family system has the greatest impact on student development (Liu and Meng, 2009), which is specifically influenced by relationships between family members (Xu et al., 2019; Conger and Donnellan, 2021); for example, increased conflict between parents makes children more likely to have conflicts with other students at school (Ingoldsby et al., 2001; Harold and Sellers, 2018). Family systems theory suggests that a good family environment promotes children’s physical and mental development, whereas an unfavorable one hinders it (Cox and Paley, 2003; Walsh, 2016; Minuchin, 2017). Based on the ecosystem theory, the family system has the greatest influence on student development; accordingly, good parent characterizes a favorable family environment–child relationships, can keep children emotionally stable, and can reduce problem behaviors.

However, as suggested above, Chinese parents may need help maintaining good relationships with their children and a favorable family environment. To facilitate family education that supports healthy child development, it is necessary to clarify the relationships between Chinese parents’ behaviors, especially around their children’s education, and child behavior to uncover best practices for family education. This study responds to this task. Specifically, this study enriches research in family systems theory by studying the relationship between parenting anxiety, authoritative parenting style, parent–child conflict, and child behavioral problems in the family system. A survey was conducted on 10,441 families in Hubei Province, China, to identify the intrafamilial factors influencing behavioral problems in primary and secondary school students. This study offers valuable insights for research and practice on preventing and controlling child behavioral problems and improving the quality of family education received by children aged 6–18.

## 2 Literature review and hypothesis development

### 2.1 Educational anxiety and child behavioral problems

“Parental education anxiety” refers to emotional and psychological experiences triggered by the stresses and challenges parents experience due to their children’s education, including uncertainty about their children’s future development, expectations of academic performance, and anxiety brought about by comparisons between their children and their children’s peers (Hossain and Roopnarine, 2020; Kim S. et al., 2020). While parental educational anxiety manifests differently in different countries and regions—for example, parents in some countries may be more focused on their children’s school performance, while those in others may be more concerned about their children’s social skills, creativity, and self-perception—it is a universal phenomenon (Alharbi and Al-Khateeb, 2020; Kim E. Y. et al., 2020; Shumow et al., 2020; Zhong et al., 2021). Regardless of its form, parenting anxiety can negatively impact family life and threaten children’s healthy development (Lee et al., 2020). In this study, parental educational anxiety refers explicitly to the tension, anxiety, worry, annoyance, and panic that parents experience due to the uncertainty of their educational outcomes, such as fears about their children falling “behind in education,” the stress of being “educationally overburdened,” the stress of “educational backwardness,” and worries over “educational incompetence” (Chen and Xiao, 2014). Notably, parental education anxiety is a kind of state anxiety that exists during their children’s education and fluctuates with changes in their children’s performance or other educational factors. When a child is removed from an educational scenario, parental educational anxiety may decrease or even disappear (Yin et al., 2022).

The American educational community defines “problem behaviors” in children and adolescents as issues with learning and interpersonal relationships, inappropriate actions and emotions, generalized emotional depression and distress, and learning-related physical symptoms (American Psychological Association, 2015; National Association of School Psychologists, 2018). Meanwhile, in China, “problem behaviors” are more generally recognized as behaviors that are abnormal for the child’s age, both in terms of severity and duration (General Office of the State Council of the People’s Republic of China, 2021). Achenbach and Edelbrock (1978) divided behavioral problems into three major categories: internalizing, externalizing, and mixed behavior problems, and developed the Achenbach Child Behavior Checklist (CBCL) in 1987 from these categories. Based on this, this study defines “child problem behaviors” as a variety of behaviors that are common and detrimental to the development of character and physical and mental health. These behaviors include externalizing and internalizing problem behaviors, which are non-adaptive behaviors that violate social norms, including aggression, violence, discipline issues, and other problem behaviors. Negative emotions and tendencies, including anxiety, depression, and withdrawal, accompany internalizing problem behaviors.

Several studies have shown that parents’ educational anxiety negatively impacts their children’s behaviors. This may be because such parents may be more inclined to use punitive methods rather than positive guidance and rewards to change their children’s behaviors. These parents tend to focus more on their children’s surface behaviors and lack adequate attention and support for intrinsic issues, such as emotion management (Wang and Zhou, 2022) and social skills (Beelmann and Lösel, 2021; Scharf and Mayseless, 2021). However, reasonable educational anxiety can promote children’s learning and development (Yang and Xia, 2018; Haßler et al., 2020). The key is to balance parental expectations and concerns with support and encouragement for children’s autonomous development and creativity. Based on the above discussion, the first research hypothesis of this study is proposed.

*Hypothesis 1*: Parental educational anxiety affects children’s behavioral problems.

### 2.2 Parent–child conflict and child behavioral problems

The parent–child relationship is grounded in blood relationships and a common living environment and involves nurturing, parenting, and support (Schoppe-Sullivan et al., 2020; Baucom et al., 2021). It is the most basic relationship between an individual and their family and the first interpersonal relationship to which an individual is exposed (Ling et al., 2018). Notably, the parent–child relationship reflects the degree of emotional connection between parents and their children (Furman and Buhrmester, 1985).

Most leading research on parent–child relationships outside of China are a few decades old. Authoritative academic definitions of parent–child relationships highlight different aspects of the relationship. For example, Belsky (1984) defines the relationship as “an intimate, emotional bond involving parental care, support, and encouragement of the child’s growth.” Meanwhile, Darling and Steinberg (1993) describe the parent–child relationship as a two-way interactive process that involves parental control and support of the child and the child’s responses and reactions to their parent. Baumrind (1991) established that the relationship also includes parents’ norms for and demands of their children and their responses to the behaviors exhibited by their children. These studies provide valuable insights into the importance and impact of parent–child relationships and guidance on how to improve them.

Parent–child conflict (PCC) refers to tensions, arguments, and disagreements between parents and their children, usually involving issues of behavior, expectations, values, power, and control (Laursen et al., 2021). Various factors, such as personality differences, cultural differences, educational styles, and miscommunication, may cause such conflicts. Regardless of the cause, such conflicts may profoundly impact the development and growth of children and youths, requiring appropriate interventions and solutions (Davies and Martin, 2021).

Scholars have suggested that the parent–child relationship is rooted in multiple factors, including parenting style, children’s behaviors and reactions, and the family environment. Good parent–child relationships cultivated by a positive family environment can prevent and reduce child behavioral problems (Robinson and Hoyt, 2021; Lee et al., 2022). On the other hand, poor parent–child relationships, such as those caused by parent–child conflict, can cause problem behaviors in children (Skibbe et al., 2021; Kim and Kim, 2022). These findings remind us that parents should minimize conflict with their children, improve their relationships with them, and help them develop positive emotional management and problem-solving skills to nurture their healthy development. Based on the above discussion, the second research hypothesis of this study is proposed.

*Hypothesis 2*: Parent–child conflict affects child behavioral problems.

### 2.3 Educational anxiety and parent–child conflict

Educational anxiety and parent–child conflict are closely related (Kim and Lee, 2022). Parents have high expectations for their children’s academic achievement and development. However, stress and anxiety are common trends in the process of reaching these expectations, causing parents to be overly demanding and critical of their children’s performances, which can increase family tensions and parent–child conflict. In addition, asymmetries between parents’ educational expectations and standards and their children’s abilities and interests may lead to conflict. Suppose a child feels they cannot meet their parents’ expectations or are forced to do something they are not interested in. In that case, they may develop negative emotions, further exacerbating parent–child conflict. A study examining the effect of parents’ educational anxiety on their children’s academic achievement showed that parents’ excessive focus on academic achievement and desire for their children to succeed academically are psychological control behaviors that may lead to educational anxiety by causing parent–child conflict (Liu X. et al., 2021; Wu et al., 2022). Therefore, parents need to be aware of their parenting styles and avoid excessive psychological control behaviors to avoid conflict with their children and youths to reduce educational anxiety.

In addition, research has shown that parent–child conflict mediates educational expectations and educational anxiety. When parents have high educational expectations for their children that do not match their children’s abilities, parent–child conflict is triggered, which can increase educational anxiety (Yin et al., 2021). Scholars have also explored the role of parent–child conflict in mediating the relationship between parenting stress and child behavior problems; specifically, they found that parents with higher parenting stress tend to have more conflicts with their children, which leads to more behavioral problems in the children (Fang et al., 2018). Additionally, researchers have reported that parent–child conflict mediates the relationship between parental attachment style and adolescent depressive symptoms (Akin, 2020). Based on the above discussion, we propose the following hypotheses.

*Hypothesis 3*: Educational anxiety triggers parent–child conflict.

*Hypothesis 4*: Parent–child conflict mediates the relationship between educational anxiety and child behavioral problems.

### 2.4 Moderating role of parenting style

In the 1960s, Baumrind (1966, 1967) introduced the most influential and far-reaching work on the dimensions of parenting styles. Specifically, based on a study in which she used naturalistic observation and interview methods to study more than 100 families with preschool-aged children, she reported that the main differences between parenting styles are reflected by two dimensions of parenting style: meeting needs and insisting on demands. The need-satisfying dimension refers to parents’ conscious efforts to foster self-discipline and self-confidence in their children by supporting their children’s behavior. The insistence dimension refers to making children comply with family elders’ decisions through orders, supervision, discipline, and punishment. Based on the differences in the intensity of these two dimensions, Baumrind identified four parenting styles: authoritative (highly demanding, highly satisfying), coddling (lacking in demand, highly satisfying), authoritarian (highly demanding, lacking in satisfaction), and neglectful (lacking in demand, dissatisfaction). This study uses Baumrind’s definition of an authoritative parent–child relationship. The authoritative type, also known as the democratic type, refers to parents who demonstrate a highly demanding parenting style—setting reasonable limits and monitoring their children’s activities and behaviors through guidelines—and a highly responsive style—nurturing their children’s sense of self-determination and individuality through warm, reasoned, and democratic communication. These parents are more likely to reason with their children or correct their behavior in supportive ways if children fail to follow their parents’ guidelines. Democratic parents balance their demands and satisfaction with those of their children to encourage their children to be independent while still complying with family rules (Baumrind, 1966, 1967, 1971). This parenting style promotes children’s autonomy and helps them to develop positive behavioral patterns.

Authoritative parenting plays a moderating role in various outcomes associated with child and adolescent development. A study found that authoritative parenting moderated the relationship between parental academic expectations and adolescent academic achievement (Wang et al., 2022). Meanwhile, another reported that authoritative parenting moderates academic self-efficacy and achievement (Hayek et al., 2022). Additionally, researchers have found that authoritative parenting moderates the relationship between cyberbullying victimization and perpetration among Pakistani children and youths (Farooq and Awan, 2021). These findings emphasize the importance of authoritative parenting for positive development. Based on the above discussion, the fifth research hypothesis of this paper is proposed.

*Hypothesis 5*: Authoritative parenting moderates the relationship between educational anxiety and parent–child conflict that impacts child behavioral problems.

## 3 Materials and methods

### 3.1 Research tools

SPSS 23.0 and AMOS 24.0 were used to process the data in this study. Reliability analysis was performed using SPSS 23.0; the total scale reliability was 0.842, which is good and reliable. Exploratory factor analysis KMO was 0.894, and KMO greater than 0.8 is considered suitable for factor analysis. Large-scale questionnaires containing the following content were created for unified data management.

#### 3.1.1 Parental education anxiety questionnaire

The Parental Education Anxiety Questionnaire was adapted from a questionnaire compiled by Xia et al. The questionnaire includes 12 items, reaching across the six dimensions of academic anxiety, physical anxiety, safety anxiety, psychological anxiety, future anxiety, and teacher anxiety (Yin et al., 2022). This study divided nine topics into academic achievement anxiety and non-academic achievement anxiety. The questionnaire adopted a five-point Likert scoring method; the higher the total score, the higher the degree of educational anxiety. Through confirmatory factor analysis, it was found that *x*^2^/*df* = 6.98, RMSEA = 0.11, RMR = 0.08, NFI = 0.88, RFI = 0.83, IFI = 0.90, TLI = 0.86, and CFI = 0.90 and the internal consistency of the selected items was 0.82, indicating that the reliability and validity of the questionnaire were consistent.

#### 3.1.2 Parent–child relationship scale

The Parent–Child Relationship Scale was adapted from the Parent–Child Relationship Scale compiled by Pianta in 1992. The Chinese version of the scale was developed after a translation and revision by Xiao et al. The scale contains 26 items divided into the three dimensions of intimacy, conflict, and dependence (Xu, 2022). This study selected all nine questions in the intimacy dimension and 12 questions in the conflict dimension. The questions in the conflict dimension were reverse-scored and then summed with the scores in the intimacy dimension. The higher the score, the better the parent–child relationship (Zu et al., 2022). This measure adopted a Likert five-point scale. The confirmatory factor analysis revealed that *x*^2^/*df* = 4.70, RMSEA = 0.08 (RMSEA values greater than 0.08 can be described as having moderate goodness-of-fit), RMR = 0.13, NFI = 0.84, RFI = 0.82, IFI = 0.87, TLI = 0.85, and CFI = 0.87, and the internal consistency reliability of the selected items was 0.78, indicating that the reliability of the scale meets the psychometric requirements.

#### 3.1.3 Parental authority questionnaire

The Parental Authority Questionnaire, compiled by Buri in 1991, was revised into Chinese by Zhou et al., who confirmed its reliability and validity. The revised PAQ finally consists of 26 items, including 11 in the authority dimension, 10 in the autocracy dimension, and five in the *laissez-faire* dimension (Shang, 2015). This study selected five questions from the PAQ: two in the authoritative dimension and three in the authoritarian dimension. The responses to the questionnaire ranged from very different to very agreeable, based on a five-point score. Through confirmatory factor analysis, it was found that *x*^2^/*df* = 21.59, RMSEA = 0.20, RMR = 0.18, NFI = 0.81, RFI = 0.53, IFI = 0.82, TLI = 0.54, and CFI = 0.82, indicating that the validity of the questionnaire met the requirements of psychometrics. The reliability of internal consistency in the authoritative dimension was 0.77, and that in the authoritarian dimension was 0.48.

#### 3.1.4 Self-administered child behavior problem scales

The CBCL scale, developed by Achenbach and Edelbrock in 1976, was revised in 1983 as the Child Behavior Scale for Parents. This scale is used to rate children’s behavioral, emotional, and social competence (Rey et al., 1992). Moreover, the scale is widely revised and used by Chinese authors (Huang et al., 2022b). In our study, the self-made Child Behavior Problem Scale was subjected to a validated factor analysis, RMR = 0.09, NFI = 0.94, IFI = 0.94, CFI = 0.94, GFI = 0.96, AGFI = 0.89, Root Mean Square Residual (RMR) <0.1, Normed Fit Index (NFI) >0.9, Incremental Fit Index (IFI) >0.9, Comparative Fit Index (CFI) > 0.9, Goodness-of-Fit Index (GFI) > 0.9, Adjusted Goodness-of-Fit Index (AGFI) close to 0.9, and Tucker-Lewis Index (TLI) close to 0.9. The scale meets the validity requirements of psychometrics. The self-administered behavioral scales in this study refer to the Achenbach Child Behavior Scale (CBCL).

### 3.2 Research object

The participants in this survey were parents of students (children and youth aged 6–18 years) in Hubei Province. We divided them into three groups according to their children’s school age. A total of 10,441 questionnaires were collected. A true understanding of the problems in home education and parents’ confusion in home education is a prerequisite for providing targeted advice and assistance to parents. Based on this starting point, we conducted a more comprehensive analysis of the administrative planning of Hubei Province and thoroughly pre-planned the representativeness of the sample. In addition, it was estimated that a large sample provides more analyzable space for our study as the problems faced by families in different geographical areas may not be identical. Therefore, to create a sample representative of Hubei Province, the sample extended across all cities and states in Hubei Province, radiating to both rural and urban areas. To conduct the survey, we first selected schools based on their distribution across Hubei Province—specifically, we selected primary and secondary rural and urban schools that were representative of the southeast, northwest, and north of the province. We then contacted teachers in the selected schools and, through the teachers, created balanced samples of students and parents in all grades. Next, we asked each teacher to encourage approximately 300 parents to complete the questionnaire. The parents completed the questionnaires online. The characteristics of the sample are listed in Table 1.

**Table 1:** Basic information derived from the sample.

Name	Option	Percentage
Location	Village	18.88%
	Cities and towns	21.50%
	County town	12.34%
	City	47.28%
Father’s highest level of education	Junior high school and below	44.76%
	High school/technical secondary school	30.58%
	University and college	12.37%
	Undergraduate	10.43%
	Graduate and above	1.86%
Annual household income	Below 50,000 yuan	34.63%
	50,000 to 100,000 yuan	34.30%
	100,000 to 150,000 yuan	14.12%
	150,000 to 200,000 yuan	7.97%
	200,000 to 300,000 yuan	4.99%
	300,000 to 400,000 yuan	2.07%
	400,000 yuan and above	1.93%
Identity	Father	24.49%
	Mother	70.81%
	Grandparents or maternal grandparents	2.85%
	Other	1.85%
Mother’s highest level of education	Junior high school and below	49.41%
	High school/technical secondary school	28.07%
	University and college	12.39%
	Undergraduate	8.80%
	Graduate and above	1.32%
Marital status	Married	90.71%
	Single (including divorced or widowed)	6.56%
	Remarried	1.74%
	Other	0.99%
Number of children	One	36.41%
	Two	57.33%
	Three	5.63%
	4 or more	0.62%

### 3.3 Data object

SPSS23.0 and AMOS23.0 were used to analyze the data. SPSS23.0 was used for conducting descriptive statistical analysis and the PROCESS macro program was used to test the mediating and moderating effects. After the normality test, it was found that the skewness value of educational anxiety data = 0.071 (standard error 0.105), Z-score = 0.671, kurtosis value = 0.127 (standard error = 0.210), and Z-score = 0.666. The Z-score were all between ±1.96, which conformed to the normal distribution. This study establishes a model with educational anxiety as the independent variable, child behavioral problems as the dependent variable, parent–child conflict as the mediating variable, and authoritative parenting style as the moderating variable to explore the relationship between authoritative parenting style, educational anxiety, parent–child conflict, and child behavioral problems.

## 4 Results

This study utilized anonymous measurements, reversed scores on certain items, and employed other measures to account for common method deviations. Harman’s single-factor test was used to test the common method deviations of the collected data. Six factors with characteristic roots greater than one were extracted from the rotational exploratory factor analysis results, and the maximum factor variance explanation rate was 23.97%. More than one factor had a characteristic root greater than 1, and the maximum factor variance was less than 40%; therefore, there was no serious common method deviation.

Table 2 shows that the average parent–child relationship score was 4.32. Taking the theoretical median of 3.5 as the reference point, the single sample T-test showed a significant difference between the parent–child relationship score and the median (*t* = 86.447, *p* < 0.001), indicating that the parent–child relationship was significantly higher than the theoretical median level. Average intimacy was 4.41 (*t* = 99.984, *p* < 0.001) and average conflict was 2.74 (*t* = −81.911, *p* < 0.001), indicating that most parent–child relationships are more intimate than conflictual. Regarding educational anxiety, the average value was 2.91. Taking the theoretical median value of 3 as the reference point, the single sample T-test showed a significant difference between the educational anxiety score and the theoretical median value (*t* = −11.902, *p* < 0.001), indicating that overall educational anxiety was significantly lower than the theoretical median value.

**Table 2:** Educational anxiety, parent–child relationship, and child behavioral problems.

	Minimum value	Maximum	Average value (m)	Standard deviation (S.D.)	Theoretical median	T
Parenthood	1.10	6.00	4.32	0.84	3.5	86.447***
Intimacy	1.00	6.00	4.41	0.93	3.5	99.984***
Conflict	1.00	6.00	2.74	0.94	3.5	−81.911***
Educational anxiety	1.00	5.00	2.91	0.75	Three	−11.902***
Academic achievement anxiety	1.00	5.00	3.28	0.92	Three	31.186***
Non-academic achievement anxiety	1.00	5.00	2.62	0.79	Three	−49.138***
Child behavioral problems	1.00	6.00	2.58	0.99	3.5	−95.174***
Authoritative type	1.00	6.00	4.41	1.14	3.5	80.938***

*p < 0.05, **p < 0.01, ***p < 0.001.

Moreover, the standard deviation of 0.75 showed that the level of educational anxiety was discrete; that is, the level of educational anxiety significantly varied. The average academic achievement anxiety was 3.28, significantly higher than the theoretical median (*t* = 31.186, *p* < 0.001). Meanwhile, the average non-academic achievement anxiety was 2.62, significantly lower than the theoretical median (*t* = −49.138, *p* < 0.001). These results suggest that parents were concerned about their children’s achievements and had educational anxiety. The average score for child behavioral problems was significantly lower than the theoretical median (*t* = −95.174, *p* < 0.001), suggesting that the participants did not have children with very serious behavioral problems. The authoritative parenting style was significantly higher than the theoretical median (*t* = −80.938, *p* < 0.001), indicating that most participants had authoritative parenting styles (Table 2).

Statistical analysis revealed that child’s age was significantly correlated only with the parent–child relationship, which showed a low level of slippage as the child’s age increased (*r* = −0.05, *p* < 0.001). There was no significant correlation between age and educational anxiety, children’s behavioral problems, and the level of parental authority in parenting; that is, parental educational anxiety, children’s behavioral problems, and the level of parental authority are not subject to change with the child’s age.

However, there were significant differences in parents’ educational anxiety, parent–child relationship, children’s behavioral problems, and the degree of authoritative parenting received with regard to children’s place of residence. Urban children were significantly better than rural students in terms of parent–child relationship (*t* = 4.88, df = 7,843, *p* < 0.001), the degree of authoritative parenting received (*t* = 3.54, df = 10,436, *p* < 0.001), and closeness to their parents (*t* = 3.94, df = 10,436, *p* < 0.001). Rural students were significantly better than urban students in terms of educational anxiety in academics (*t* = −7.94, df = 10,436, *p* < 0.001), non-academic dimensions (*t* = −8.84, df = 10,436, *p* < 0.001), conflict with parents (*t* = −3.70, df = 10,436, *p* < 0.001), and problematic behaviors (*t* = 5.52, df = 10,436, *p* < 0.001).

We selected samples of parents from different places and age groups to study the representativeness of the samples. A present, in various regions of China, students of different ages have very similar problems. They face significant pressure to study from their parents. This situation is especially prominent in non-urban than urban regions, and this study explored the impact of such a dysfunctional parent–child relationship on children and suggested ways to cope with it.

### 4.1 Correlation between educational anxiety, parent–child relationship, and child behavioral problems

A Pearson correlation analysis showed that educational anxiety, the parent–child relationship, and child behavioral problems were correlated in pairs. The correlation coefficient between educational anxiety and the parent–child relationship is −0.278**, and the correlation coefficient between educational anxiety and child behavioral problems is 0.277**, indicating that educational anxiety was positively correlated with child behavioral problems; this validates Hypothesis 1—that is, it establishes that parental educational anxiety affects the behavioral problems of primary and secondary school students. Further, we also found that educational anxiety is negatively correlated with parent–child relationships; this validates Hypothesis 2—that is, it confirms that parental educational anxiety affects the quality of primary and secondary school students’ relationships with their parents. Meanwhile, the correlation coefficient between the parent–child relationship and child behavioral problems is −0.547**, indicating that the parent–child relationship is negatively correlated with child behavioral problems.

### 4.2 Correlations between education anxiety, parent–child relationship, and child behavioral problems across different dimensions

A Pearson’s correlation analysis showed that the correlation coefficients for each dimension of educational anxiety and child behavioral problems were 0.241** and 0.244**, respectively, revealing a significant positive correlation between each dimension of educational anxiety and child behavioral problems. The correlation coefficient between the intimacy dimension of the parent–child relationship and child behavioral problems was −0.173**, showing a significant negative correlation between these factors. The correlation coefficient between the conflict dimension of the parent–child relationship and child behavioral problems was 0.613**, showing a significant and close positive correlation between these factors; this validates Hypothesis 3—that is, it reveals that parent–child conflict can trigger behavioral problems in children. The correlation coefficients between each dimension of educational anxiety and the intimacy dimension of the parent–child relationship were − 0.073** and − 0.050**, respectively, indicating weak negative correlations. The correlation coefficients of each dimension of educational anxiety and the conflict dimension of the parent–child relationships were 0.255** and 0.233**, respectively, showing a significant positive correlation between these factors (Table 3).

**Table 3:** Correlations among educational anxiety, parent–child relationship, and child behavioral problems.

	One	Two	Three	Four	Five	Six	Seven
1. Education anxiety	One						
2. Academic anxiety	0.868**	One					
3. Non-academic achievement anxiety	0.888**	0.542**	One				
4. Parent–child relationship	−0.278**	−0.262**	−0.228**	One			
5. Intimacy	−0.069**	−0.073**	−0.050**	0.690**	One		
6. Conflict	0.277**	0.255**	0.233**	−0.845**	−0.195**	One	
7. Child behavioral problems	0.277**	0.241**	0.244**	−0.547**	−0.173**	0.613**	One

*p < 0.05, **p < 0.01, ***p < 0.001.

### 4.3 Testing the mediating effect of parent–child relationships

In this study, Model 4 in the PROCESS macro program was used to test the mediating effect of parent–child conflict in the relationship between educational anxiety and child behavioral problems. As Table 4 shows, educational anxiety significantly positively predicted child behavioral problems (*β* = 0.352, *p* < 0.001), indicating that the greater the parents’ educational anxiety, the more serious the child’s behavioral problems are likely to be. Educational anxiety significantly positively predicted conflict (*β* = 0.342, *p* < 0.001), indicating that the greater the parents’ educational anxiety, the greater the conflict between parents and children; this validates Hypothesis 4—that is, it shows that conflictual parent–child relationships mediate the association between educational anxiety and child behavior problems. When educational anxiety and parent–child conflict simultaneously predicted child behavioral problems, they both significantly positively predicted them (*β* = 0.145, *p* < 0.001, *β* = 0.605, *p* < 0.001), indicating that conflict strongly influences child behavioral problems when educational anxiety and parent–child conflict co-exist.

**Table 4:** The mediating effect of conflict between educational anxiety and child behavioral problems.

Outcome variable	Predictor variable	*R*	*R^2^*	*F*	*Β*	*Se*	*T*
Child behavioral problems	Educational anxiety	0.292	0.085	121.278***	0.352	0.013	28.08***
Conflict	Educational Anxiety	0.284	0.08	114.006***	0.342	0.012	28.513***
Child behavioral problems	Educational anxiety	0.626	0.391	744.774***	0.145	0.011	13.679***
	Conflict				0.605	0.008	72.422***

* stands for *p* < 0.05, **p < 0.01, **p < 0.001.

Furthermore, the bootstrap program method was used to test the mediating effect of conflict between educational anxiety and child behavioral problems. The number of repeated random samplings of bootstrap samples was set to 5,000 times. As shown in Table 5, the direct effect value of educational anxiety on child behavioral problems is 0.145, and the 95% confidence interval is [0.125, 0.166], indicating that the direct effect is significant. The mediating effect of conflict between educational anxiety and child behavioral problems was 0.207, and the 95% confidence interval was [0.189, 0.226], with an effect size of 0.59, which showed that the mediating effect of parent–child conflict was significant. Therefore, parent–child conflict partially mediates the relationship between educational anxiety and child behavioral problems. This suggests that while educational anxiety can directly affect child behavioral problems, it can also affect them through parent–child conflicts.

**Table 5:** Decomposition table of total effect, direct effect, and intermediary effect.

	Effect	BootSE	*t*	BootLLCI	BootULCI
Total effect	0.352	0.013	28.080***	0.328	0.377
Direct effect	0.145	0.011	13.679***	0.125	0.166
Indirect effect	0.207	0.009		0.189	0.226

*p < 0.05, **p < 0.01, ***p < 0.001.

### 4.4 An adjusted mediating effect test

**Figure 1:**
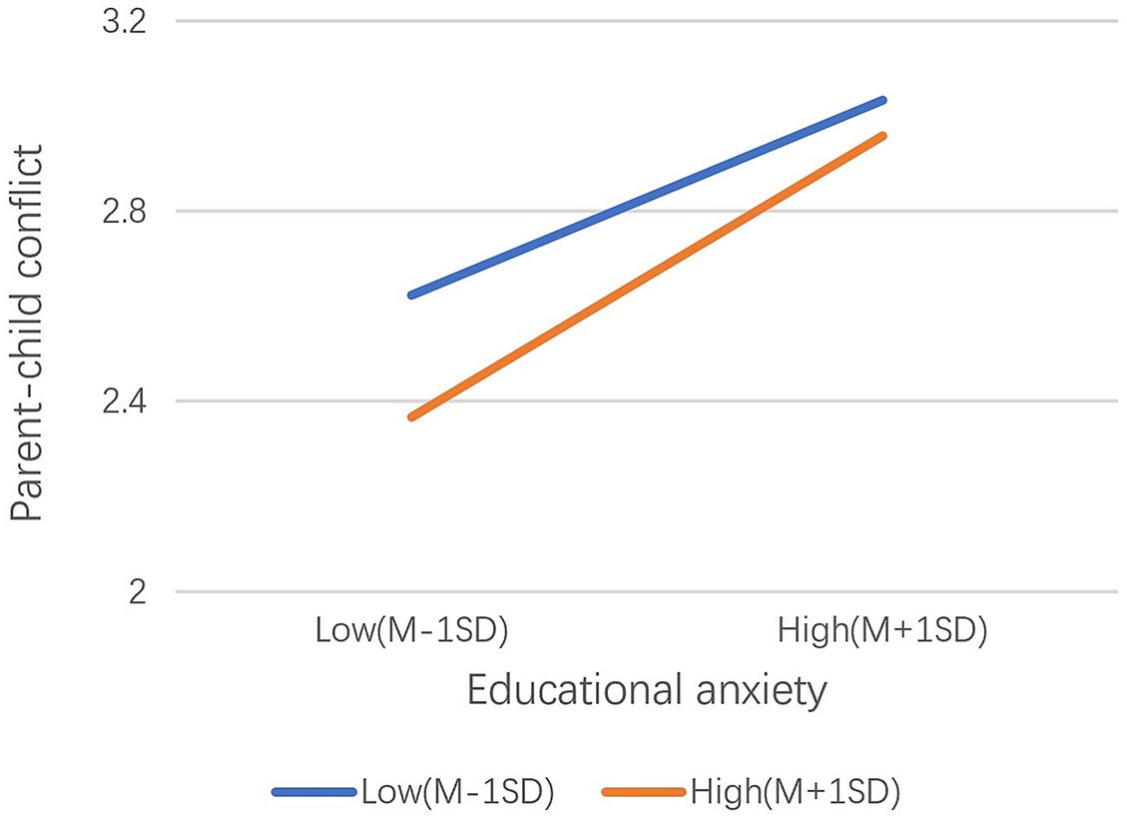
Moderating effect of an authoritative parenting style on the relationship between educational anxiety and parent–child conflict.

**Figure 2:**
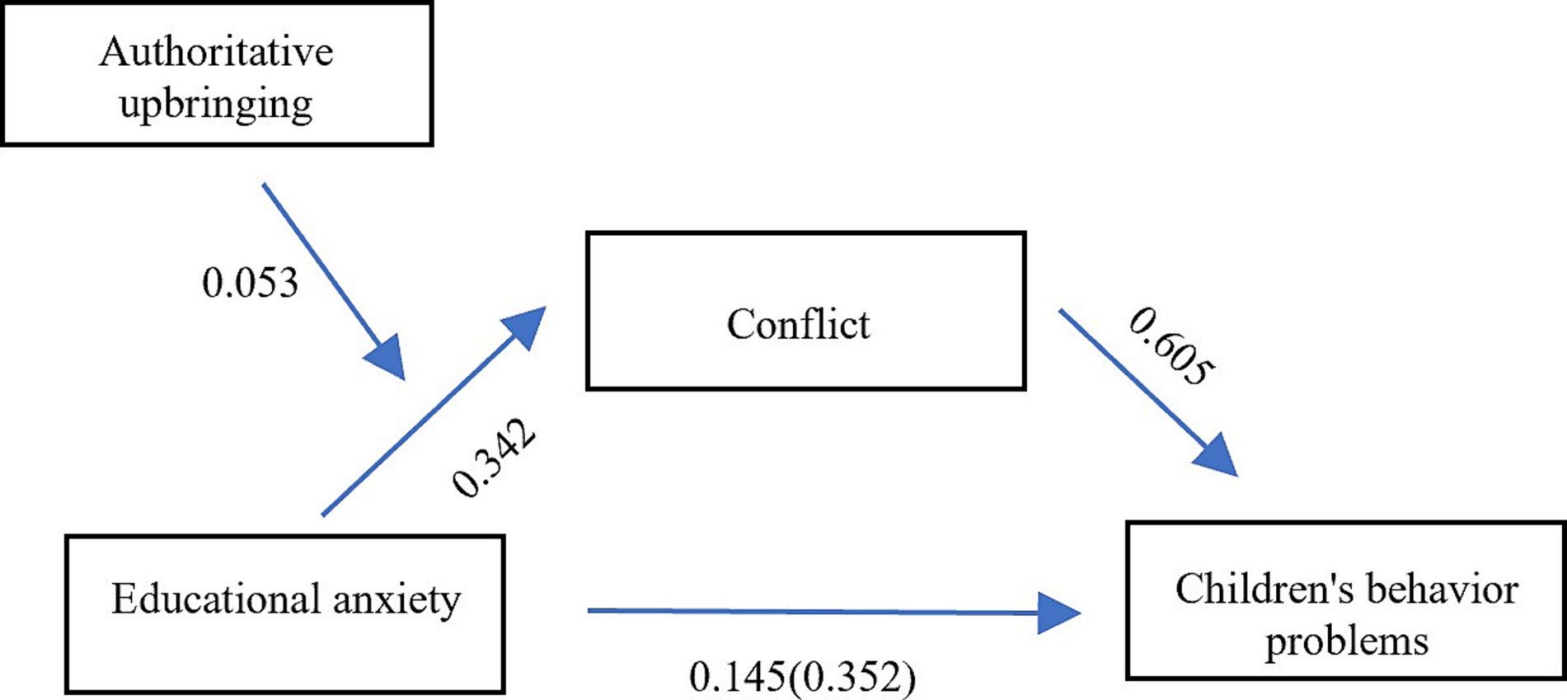
Research model diagram.

Model 7 (adjusted for the first half of the mediation) in the PROCESS macro program was used to test the mediated effect. As Table 6 shows, educational anxiety has a significant moderation effect on parent–child conflict (*β* = 0.335, *p* < 0.001), authoritative parenting style has a significant negative predictive effect on conflict (*β* = 0.072, *p* < 0.001), and the interaction between educational anxiety and authoritative parenting style has a significant predictive effect on conflict (*β* = 0.053, *p* < 0.001). To clarify the essence of the interaction between educational anxiety and authoritative parenting style, authoritative parenting style was divided into high and low groups according to a standard deviation of plus or minus one, and the influence of educational anxiety on conflict at different levels of authoritative parenting style was investigated using a simple slope test. As Figure 1 shows, a low level of authoritative parenting (M-1SD) had a significant moderation effect on child behavioral problems (simple slope = 0.274, *t* = 16.676, *p* < 0.001). Meanwhile, a high level of authoritative parenting style (M + 1SD) had a significant moderation effect on child behavioral problems (simple slope = 0.396, *t* = 25.021, *p* < 0.001); this confirms Hypothesis 5—that is, it evidences that parenting styles play a moderating role in the relationship between parental educational anxiety and parent–child conflict; accordingly, parenting styles consequently affect how this relationship shapes child behavioral problems (Figure 2). Although the moderation effect of educational anxiety on conflict continues to increase as the authoritative parenting style improves, the level of parent–child conflict decreases, suggesting that authoritative parenting can weaken the moderation effect of educational anxiety on parent–child conflict. Table 7 shows the results of further testing of the moderating effect of authoritative parenting style, revealing the influence of educational anxiety on child behavioral problems through parent–child conflict and the confidence interval. The mediating model constructed in this study holds an effect size of 0.22 (index = −0.032, bootstrap = 0.008, 95% CI = [0.016, 0.049]) (Table 6).

**Table 6:** Adjustment effect test.

Variable	Conflict				
	Β	Se	T	Llci	Ulci
Income	−0.017	0.007	−2.619**	−0.03	−0.004
Cities and towns	−0.001	0.028	−0.034	−0.056	0.054
County town	−0.055	0.033	−1.695	−0.119	0.009
City	−0.008	0.025	−0.338	−0.058	0.041
Father	−0.111	0.066	−1.683	−0.24	0.018
Mother	−0.028	0.067	−0.423	−0.16	0.103
Grandparents or maternal grandparents	−0.086	0.083	−1.026	−0.249	0.078
Educational anxiety	0.335	0.012	28.055***	0.312	0.359
Authoritative type	−0.072	0.008	−9.317***	−0.088	−0.057
Educational anxiety * authoritative type	0.053	0.009	5.596***	0.034	0.072
R^2^			0.092		
F			105.128***		

*p < 0.05, **p < 0.01, ***p < 0.001.

**Table 7:** Mediating effect of regulation.

Authoritative type	Effect	BootSE	BootLLCI	BootULCI
−1.145	0.166	0.013	0.141	0.191
0	0.203	0.009	0.186	0.221
1.145	0.240	0.013	0.214	0.266
Regulated intermediary	0.032	0.008	0.016	0.049

## 5 Discussion

In China, regardless of the age or location of students, the primary pressure they face at home is academic achievement, and the source is their parents (Hong and Liu, 2021). This study revealed the influence of educational anxiety on child behavioral problems. After adding parent–child conflict as an intermediary variable, it was found that educational anxiety not only directly affects child behavioral problems but also indirectly affects them by increasing parent–child conflict, which partially mediates their relationship.

First, the complex relationships among educational anxiety, the parent–child relationship, and child behavioral problems reveal the current state of family education in China. Most parents attach excessive importance to their children’s academic performance (Liu G. et al., 2021; Chang et al., 2022); parents place their children’s academic performance as second in importance only to their health. Because many Chinese parents rigidly require their children to perform well academically, their children’s lives are centered on studying and are, therefore, very monotonous. When parents pass their educational anxiety on to their children, their children may experience much pressure and accordingly evaluate themselves and others based on their academic performance. Children without strong learning abilities who grow up in such an environment often feel relatively high levels of anxiety and discomfort. They may not feel that they are loved within their families. Second, the higher the parents’ educational anxiety for their children, the higher their educational requirements for their children, which increases the likelihood of creating a depressed family environment and putting children under more pressure. Eventually, these trends worsen the parent–child relationship, increase parent–child conflict, and restrict the child’s development, increasing the likelihood that the child will have behavioral problems (Liu G. et al., 2021). Finally, good parent–child relationships have a long-term protective effect on child development. Authoritative parenting mediates this effect; children who grow up in a family with a positive approach to education have fewer conflicts with their parents and are less prone to behavioral problems (Yu, 2010).

These findings closely relate to China’s current educational context and family education atmosphere, which may differ in other countries. First, it is notable that China has a large population, which has created intense competition for limited resources. In the Chinese context, individuals have been brought up with the idea that parents are the first persons responsible for their children’s poor educational outcomes and that their biggest failure is their own (Xia, 2016). Thus, parents feel responsible for their children’s development and may become frustrated if their children are poorly educated. Therefore, it is not surprising that most families in China today expend great efforts to educate their children (Yu et al., 2022).

Further, the average fertility rate of a family in China is currently under 2, which means that very few families have more than two children. The social phenomenon of childlessness determines that parents only have one chance to raise their children and do not want to see their children fail. This is one of the reasons for the serious educational anxiety behavior of Chinese parents. Additionally, the double educational elimination mechanism of the midterm and college entrance exams may cause Chinese parents to feel highly anxious about their children’s educational success. If a child fails the Secondary School Examination, they will have to work as a manual laborer and lead a hard life. If a child fails the College entrance examination, they will have no competitive edge and will not be able to find a well-paid job (Sun, 2022). China’s highly competitive environment may put more pressure on parents to ensure their children are well-educated, and the anxieties related to these pressures may be passed on to their children. When parents pass their educational anxiety directly to their children, tensions can emerge in the parent–child relationship, increasing the likelihood of child behavioral problems.

Meanwhile, in countries without double educational elimination mechanisms, children may not be educationally differentiated until the university level, when they will be adults who can take responsibility for their behaviors. In such contexts, parental educational anxiety may be less intense than that observed in China. Accordingly, the process revealed in this study may not be so apparent in other cultural environments. However, because parents are naturally caught up in their children’s education by virtue of raising them, they will always have some degree of anxiety about their children’s education. Therefore, this study’s finding that the education sector can improve children’s and parent’s health and strengthen their mutual harmony by providing adequate support to parents may also be applicable in these other contexts.

The results of this study also underscore that parents should provide their children with care, understanding, support, encouragement, a suitable living environment, appropriate educational methods, and positive parenting styles. Excessive anxiety and parent–child conflicts are not conducive to healthy child development. However, there are some existing barriers to this work. Brown and Brenner’s ecosystem theory (Strauss, 2021), mentioned in the introduction, maintains that an environment has a significant influence on individual development—specifically, the microsystem, mesosystem, exosystem, macrosystem, and ephemeral system all influence individual development to different degrees. Considering the family educational problems in Hubei Province, the ecosystem theory suggests that we can make efforts in the following aspects. First, at the macrosystem level, we should update existing conceptions of a “successful” parent. Many parents still educate their children according to traditional methods, believing that if their children’s academic performance is excellent, nothing else requires attention—as a result, elements such as child mental health, parent–child communication, and interpersonal interactions are often neglected.

Parental leadership at the socio-cultural level is necessary. Second, at the exosystem level, the education sector should better support family education. Parents’ attitudes toward their parenting and effort levels are both exosystemic factors. Chinese parents want to guide their children’s education but also want to be guided in this work themselves. Notably, they are impatient and always hope for a one-time cure for all problems. The education department can help parents shape their children’s education by offering systematic support and targeted guidance. Third, children’s education should be actively promoted at the mesosystemic level. Effective cooperation among multiple microsystems can result in positive interactions with negative outcomes. A student’s frustration with learning in school, coupled with their parents’ scolding at home, is a classic negative interaction that can only worsen the problem. The student’s frustration in learning can be supported by warmth from their family, which can solve their behavioral problems. Fourth, at the microsystem level, guidance for children should be implemented. Families and schools are the microsystems where elementary, junior high, and secondary school students live. How parents interact with their children directly affects the atmosphere of the family system and children’s growth. When parents encourage each other in their roles in child-rearing, each one will parent more effectively. Further, when parents and children encourage each other, and teachers and students support each other, the microsystems of children’s lives are healthier.

Today, schools offer guidance by means of traditional forms of family education through home visits and parent–teacher conferences; however, it is not enough to rely solely on traditional schools to guide family education. The sampling survey and quantitative evaluation conclusions suggest that a family education guidance system could be rebuilt based on the following aspects.

### 5.1 Effective social supply

First, community family education service sites should be established. This study found that most parents’ problems include not knowing how to raise their children, the conflict between maternal and paternal approaches, intergenerational differences in family education, and shortcomings in the child’s psychology. By setting up community family education service sites and organizing regular parent exchange meetings, parents of different age groups can share their experiences with raising their children, learn from each other’s experiences, and continuously improve their educational methods (Liu et al., 2022). Individualized activities should also be offered for families with different needs to provide them with one-on-one counseling. For example, families with intergenerational differences in family education could be offered family education guidance services to help them better understand their children and effectively co-parent; families with conflicting educational concepts could be provided with professional guidance on different parenting styles suitable for their children’s physical and mental characteristics to avoid conflicts between parents (which can affect parent–child relationships) and create a good family atmosphere.

Second, a platform for quality learning resources should be created. The data from this study show that most parents pay attention to their children’s educational information, which indicates that most parents have an awareness of learning and self-improvement. Therefore, a platform that offers data on family education services should be created to ensure that parents can personalize their learning in a targeted manner. Such a platform could use big data to provide guidance and resources according to the family environment, the child’s personality, age, and other characteristics to ensure that parents can efficiently carry out personalized learning (Hu and Wang, 2022). This study found that parents in Hubei Province had relatively low levels of education; therefore, providing parents with quality learning resources can help them understand the characteristics and rules of their children’s physical and mental development and thus better promote their children’s growth.

Finally, a professional team for family education guidance services should be established. Moreover, resources should be invested in developing family education research; this work must notably consider different historical and cultural characteristics and realities, the hotspots and difficult problems common to family education, and the outstanding guidance needs of special families (Dou and Qiao, 2023). This quality tripartite for family education guidance service is necessary for healthy child development.

### 5.2 Schools as the main source of guidance services

This study found that schools are considered the main body of family education guidance. Parents want to jointly and equally participate with schools and teachers to support their children’s education. Schools’ normative and professional nature may make family educational services more attractive to parents and society. Therefore, schools should actively provide targeted family education guidance services as needed. They should consider the diversity and specificity of family development, understand the composition of students and parents in schools, and provide individualized guidance for different types of families (Liang and Bian, 2022).

First, schools should encourage parental involvement in classroom activities. This study found that parental absence is a part of family education. Parental presence is important for children’s growth, and schools should keep abreast of parental presence in each family, strengthen equal cooperation between home and school, actively carry out class activities, encourage fathers to participate in increasing father–child interaction, and help children to be responsible for their learning. Regarding schools’ roles in increasing father–child interactions, schools could, for instance, encourage students to write monthly letters to their fathers in which they may write about their feelings, offer gratitude to their fathers, discuss their disagreements with or misunderstandings of their father, or ask for help. Schools can also encourage fathers to respond to these letters.

Second, schools should train teachers in family education. Teachers have the most frequent contact with and knowledge of their students’ families; therefore, they critically possess the core competencies needed to carry out educational guidance. Teachers’ competencies in family education guidance services do not come naturally but are acquired and enhanced through various targeted training and practices. Especially in pre-service education, there are few courses related to family education in teacher training colleges and universities, so there is a greater need to strengthen long-term training for schoolteachers, especially school leaders, classroom teachers, mental health teachers, and other key family education instructors and school administrators in the post-service period (Yang and Zeng, 2022).

Finally, school and community collaboration is essential. School services for family education are in their infancy in China, and it is difficult to rely on schoolteachers and internal resources alone to make a difference in the face of parents’ diverse needs and the highly specialized nature of family education guidance services. Therefore, schools must broaden their thinking, use social forces, and coordinate resources and support from all sides. On the one hand, they should integrate the resources of family education guidance services established by social organizations, such as women’s federations, and establish effective cooperation mechanisms with family education guidance centers in their regions and parent schools in their communities. On the other hand, the resources of social and professional organizations and professional institutions should be integrated to guide parents through purchasing services and conducting cooperative research to ensure the quality of family education guidance services and to broaden the types of family education guidance services (Bian et al., 2021). The government should also establish an effective mechanism for cooperation with schools.

### 5.3 Breaking through family dilemmas

The study’s results suggest that a strong family education guidance system would be needed to break through family dilemmas. First, the system should help families create a good family atmosphere. This study found that only a small number of parents thought that their children were receiving a good family education; most of them felt that they might need more social support from their schools to realize an ideal level of family education and an ideal atmosphere in which their children could be educated at home. Therefore, when providing family education guidance services, it is necessary to provide more social support and actively help parents create a positive family atmosphere.

Second, it will also be important to increase Parent–child intimacy through emotional companionship. On the one hand, parents should be more involved in their children’s daily activities, understand their children’s emotional needs, and take them to parent–child activities and sports activities as much as possible so that their children can feel their love and care. On the other hand, the government and community should promote theoretical knowledge and practical training on parental emotional support in family education. Parents with low income and education levels should be given more support and assistance, especially in rural areas. Family guidance can be flexibly used by parents with educational experience.

Finally, appropriate parenting styles can improve parent–child conflict. This study found that children who grew up with positive family values had fewer parent–child conflicts and were less likely to have behavioral problems. An authoritative parenting style can actively create an atmosphere of love, respect, and equality for their children and minimize such conflicts; therefore, parents should consider adopting this style. Parents should also keep abreast of national policies, establish correct educational concepts, reduce anxiety, and calmly communicate with their children. At the same time, parents should be offered guidelines on restricting and supervising their children’s activities and behaviors to prevent them from developing behavioral problems. Children’s sense of self-determination and individuality should be nurtured through warm, reasoned, and democratic communication to promote healthy development. Family education supply and demand should match, and the supply of family education guidance services should respond to the demand for family education guidance in a modernized context. It will be necessary to fully consider children’s learning needs and characteristics and provide targeted educational services. Families, schools, the government, and society should work together to build a family education guidance service system so that children can grow up well in an ideal family.

### 5.4 Contributions

This study contributes to existing knowledge through its research method, sample quantity, sample quality, breadth and depth, and practical value. First, this was a mixed qualitative and quantitative study. We used qualitative interviews to discover the real problems in Chinese family education and then used quantitative methods to reveal the average level and the relationship between various variables—the research both came from life and applied to life. Second, this study had a large sample size, showing the current family education situation in Hubei Province. Studying the demand for family education in the province and the current situation provides empirical support for best practices for family education in Hubei Province. Specifically, it yields practical suggestions and countermeasures to improve family education: country, society, and family.

### 5.5 Directions for future research

Research on the status of family education should move away from the self-statement scale approach. Regarding the current situation of family education, a direct questionnaire survey of parents may clarify results that may have been distorted due to various factors like social factors. Furthermore, interviews with children, teachers, and other stakeholders can deepen our understanding of family education.

## Data availability statement

The original contributions presented in the study are included in the article/supplementary material, further inquiries can be directed to the corresponding author.

## Ethics statement

The studies involving humans were approved by the Institutional Review Board of the Hubei University of Education of China. The studies were conducted in accordance with the local legislation and institutional requirements. The participants provided their written informed consent to participate in this study.

## Author contributions

LS: Conceptualization, Data curation, Formal analysis, Funding acquisition, Investigation, Methodology, Project administration, Resources, Software, Supervision, Validation, Visualization, Writing – original draft, Writing – review & editing. AL: Conceptualization, Data curation, Formal analysis, Funding acquisition, Investigation, Methodology, Project administration, Resources, Software, Supervision, Validation, Visualization, Writing – original draft, Writing – review & editing. MC: Conceptualization, Investigation, Writing – original draft. LL: Data curation, Formal analysis, Methodology, Project administration, Software, Supervision, Writing – original draft. YZ: Conceptualization, Methodology, Project administration, Validation, Writing – original draft, Writing – review & editing. AZ: Conceptualization, Formal analysis, Investigation, Writing – review & editing. PH: Data curation, Methodology, Project administration, Supervision, Validation, Writing – review & editing.

## References

[ref1] Achenbach T. M. Edelbrock C. S. (1978). The classification of child psychopathology: a review and analysis of empirical efforts. Psychol. Bull. 85, 1275–1301. doi: 10.1037/0033-2909.85.6.1275, PMID: 366649

[ref2] Akin U. (2020). Parent–child conflict as a mediator between parental attachment styles and adolescents’ depressive symptoms. J. Child Fam. Stud. 29, 1769–1777.

[ref3] Alharbi K. A. Al-Khateeb F. A. (2020). Academic procrastination, parental control, and psychological well-being of undergraduate students in Saudi Arabia: a moderated mediation analysis. Curr. Psychol. 39, 1209–1217.

[ref4] American Psychological Association (2015). Guidelines for psychological practice with transgender and gender nonconforming people. Available at: https://www.apa.org/practice/guidelines/transgender.pdf (Accessed June 2, 2023). 10.1037/a0039906 26653312

[ref5] Australian Government (2022). National Education Guidelines. Canberra: Department of Education and Training.

[ref7] Baucom K. J. Hahlweg K. Atkins D. C. Eldridge K. A. (2021). Enhancing the quality of parent–child relationships: a randomized controlled trial. J. Fam. Psychol. 35, 33–43.32437203

[ref8] Baumrind D. (1966). Effects of authoritative parental control on child behavior. Child Dev. 37, 887–907. doi: 10.2307/1126611, PMID: 38149361

[ref9] Baumrind D. (1967). Child care practices anteceding three patterns of preschool behavior. Genet. Psychol. Monogr. 75, 43–88. PMID: 6032134

[ref10] Baumrind D. (1971). Current patterns of parental authority. Dev. Psychol. 4, 1–103. doi: 10.1037/h0030372, PMID: 38187422

[ref11] Baumrind D. (1991). The influence of parenting style on adolescent competence and substance use. J. Early Adolesc. 11, 56–95. doi: 10.1177/0272431691111004, PMID: 37748417

[ref12] Bayer J. K. Sanson A. V. Hemphill S. A. (2006). Parent influences on early childhood internalizing difficulties. J. Appl. Dev. Psychol. 27, 542–6559. doi: 10.1016/j.appdev.2006.08.002, PMID: 37207732

[ref1004] Beelmann A. Lösel F. (2021). A Comprehensive Meta-Analysis of Randomized Evaluations of the Effect of Child Social Skills Training on Antisocial Development. J Dev Life Course Criminology 7, 41–65. doi: 10.1007/s40865-020-00142-8

[ref13] Belsky J. (1984). The determinants of parenting: a process model. Child Dev. 55, 83–96. doi: 10.2307/1129836, PMID: 6705636

[ref14] Bernardes J. (1999). We must not define “the family”! Marriage Family Rev. 28, 21–41. doi: 10.1300/J002v28n03_03, PMID: 38191059

[ref15] Bian Y. Yuan K. Zhang X. (2021). The current situation, challenges and policy analysis of the school-based family education guidance service system in China: results of a survey in nine provinces (cities). Chin. Educ. J. 12, 22–27.

[ref16] Bronfenbrenner U. (1992). Ecological systems theory. Philadelphia: Jessica Kingsley Publishers.

[ref17] Chang Y. Guo F. Chen Z. (2022). The influence of parents’ irrational beliefs on pupils’ behavior problems: the multiple mediating effects of parents’ anxiety and psychological control. Chin. J. Clin. Psychol. 30, 397–402. doi: 10.16128/j.cnki.1005-3611

[ref18] Chen H. Z. Xiao W. (2014). The phenomenon of “education anxiety” among Chinese parents. J. Natl Acad. Educ. Admin. 2, 18–23.

[ref20] Conger R. D. Donnellan M. B. (2021). An interactionist perspective on family relationships and adolescent development. Dev. Psychol. 57, 165–177.

[ref21] Cox M. J. Paley B. (2003). Understanding families as systems. Curr. Dir. Psychol. Sci. 12, 193–196. doi: 10.1111/1467-8721.01259, PMID: 38191398

[ref22] Danisworo D. L. Wangid M. N. (2022). The influence of family harmony and emotional regulation ability on juvenile delinquency. Eur. J. Edu. Studies 9, 2501–1111. doi: 10.46827/ejes.v9i6.4315

[ref23] Darling N. Steinberg L. (1993). Parenting style as context: an integrative model. Psychol. Bull. 113, 487–496. doi: 10.1037/0033-2909.113.3.487, PMID: 38054168

[ref24] Davies P. T. Martin M. J. (2021). Understanding parent–child conflict: contributions from neurobiology and attachment research. Fam. Process 60, 375–392.

[ref25] Ding Y. Xue H. (2022). The current situation, characteristics and influencing factors of parents’ educational anxiety—an empirical study based on 35, 162 parents. J. Cap. Norm. Univ. 5, 145–156.

[ref26] Dou Y. Qiao D. (2023). Top-level design and implementation of family education guidance service system under “homeschool-society synergy”. Chin. J. Educ. 357:34-39 + 74.

[ref27] Ermer E. Cope L. M. Nyalakanti P. K. Calhoun V. D. Kiehl K. A. (2013). Aberrant paralimbic gray matter in incarcerated male adolescents with psychopathic traits. J. Am. Acad. Child Adolesc. Psych. 52, 94–103.e3. doi: 10.1016/j.jaac.2012.10.013, PMID: 23265637 PMC3549663

[ref28] Fang Y. Zhang W. Chen X. (2018). Parent–child conflict mediates the relationship between parenting stress and child behavior problems: a longitudinal study of Chinese families. J. Fam. Psychol. 32, 116–126.

[ref29] Farooq M. S. Awan M. A. (2021). Role of authoritative parenting style in reducing cyberbullying among adolescents: moderating effect of gender. J. Child Fam. Stud. 30, 1550–1561.

[ref30] Furman W. Buhrmester D. (1985). Children’s perceptions of the personal relationships in their social networks. Dev. Psychol. 21, 1016–1024. doi: 10.1037/0012-1649.21.6.1016, PMID: 38023966

[ref31] Gao S. Bian Y. (2023). A study on the construction of a four-level system of family education guidance services in the new era - an organizational framework for Chinese-style family education guidance services. Edu. Devel. Res. 6, 18–25. doi: 10.14121/j.cnki.1008-3855.2023.06.010

[ref32] Gao Y. Hu J. Zhou L. Tu S. (2023). The relationship between parenting anxiety and adolescent emotional and behavioral problems: the mediating role of negative parenting styles. Appl. Psychol. 1, 80–88. doi: 10.20058/j.cnki.cjap.022012

[ref33] Garcia Yeste C. Morlà Folch T. Ionescu V. (2018). Dreams of higher education in the Mediterranean school system through family education. Front. Educ. 3:79. doi: 10.3389/feduc.2018.00079

[ref34] General Office of the State Council of the People’s Republic of China (2021). Guiding opinions on strengthening and improving mental health services for minors. Beijing: General Office of the State Council of the People’s Republic of China.

[ref35] Han B. Zhang L. (2015). The effect of educational expansion on juvenile delinquency. J. Northeast Normal Univ. 2, 43–48. doi: 10.16164/j.cnki.22-1062/c.2015.02.009

[ref36] Harold G. T. Sellers R. (2018). Annual research review: interparental conflict and youth psychopathology: an evidence review and practice focused update. J. Child Psychol. Psych. 59, 374–402. doi: 10.1111/jcpp.12893, PMID: 29574737

[ref37] Haßler B. Major L. Hennessy S. (2020). Learner-generated content: a framework to promote educational equity? J. Learn. Dev. 7, 67–79.

[ref38] Havigerová J. M. Haviger J. Truhlářová Z. (2013). Teacher’s subjective definition of family. Proc. Soc. Behav. Sci. 106, 2507–2515. doi: 10.1016/j.sbspro.2013.12.288, PMID: 29759070

[ref1002] Hayek J. Schneider F. Lahoud N. Tueni M. de Vries H. (2022). Authoritative parenting stimulates academic achievement, also partly via self-efficacy and intention towards getting good grades. Plos one 17:e0265595. doi: 10.1371/journal.pone.0265595 35353817 PMC8967044

[ref39] Hong X. Liu Q. (2021). Parenting stress, social support, and parenting self-efficacy in Chinese families: does the number of children matter? Early Child Dev. Care 191, 2269–2280. doi: 10.1080/03004430.2019.1702036

[ref40] Hossain Z. Roopnarine J. L. (2020). Parenting stress and practices among immigrant parents: the role of acculturation and perceptions of school climate. J. Child Fam. Stud. 29, 1033–1042.

[ref41] Hu J. Wang X. (2022). The legal framework and implementation path of family education: a study on the needs of family education in China. J. Beijing Admin. Coll. 2022, 104–112. doi: 10.16365/j.cnki.11-4054/d.2022.03.013

[ref42] Huang D. Huang Z. M. Fu G.-L. (2022b). Analysis of factors influencing parents’ willingness to provide family education guidance for primary and secondary school students: an ordered multicategorical logistic regression model. J. Contin. High. Educ. 35, 66–73.

[ref43] Huang D. Huang Z. Fu G. Ma M. (2022a). Analysis of parents’ family education guidance needs and suggestions for countermeasures under the background of “double reduction”. J. Hunan Radio Telev. Univ. 3, 30–39. doi: 10.19785/j.cnki.hnddxb.2022.03.004

[ref44] Huang Y. Liu J. Xu Y. Wang X. Guo L. Gao F. (2022). A study of the Achenbach child behavior scale (CBCL) in children with autism spectrum disorders. Chinese J. Soc. Med. 5, 571–575.

[ref45] Ingoldsby E. M. Shaw D. S. Garcia M. M. (2001). Intrafamily conflict in relation to boys’ adjustment at school. Dev. Psychopathol. 13, 35–52. doi: 10.1017/S0954579401001031, PMID: 11346051

[ref46] Kim E. Y. Cho S. Kim M. (2020). Korean mothers’ parenting style and self-efficacy on child-rearing: a moderated mediation model. Child Youth Serv. Rev. 114:105030

[ref47] Kim S. Y. Kim S. Y. (2022). Bidirectionality between parent–child conflict and adolescent problem behaviors: a longitudinal study of Korean adolescents. J. Adolesc. 96, 138–147.

[ref48] Kim E. M. Lee J. (2022). Parent–child conflict mediates the relationship between academic anxiety and behavioral problems in preschoolers. J. Child Fam. Stud. 31, 83–92.

[ref49] Kim S. Lee J. Lee H. (2020). Parental involvement and children’s academic achievement in South Korea: focusing on the mediating roles of academic motivation and self-regulation. Early Child Dev. Care 190, 434–447.

[ref50] Laursen B. Coy K. C. Collins W. A. (2021). “Adolescent-parent conflict: an overview of current research” in Handbook of adolescent psychology. eds. Lerner R. M. Steinberg L. (Hoboken: Wiley), 123–152.

[ref51] Lawrence P. J. Murayama K. Creswell C. (2019). Systematic review and meta-analysis: anxiety and depressive disorders in offspring of parents with anxiety disorders. J. Am. Acad. Child Adolesc. Psych. 58, 46–60. doi: 10.1016/j.jaac.2018.07.898, PMID: 30577938

[ref52] Lee S. J. Daniels M. H. Shin J. (2020). Understanding Korean American parents’ involvement in their children’s education and psychological well-being: the role of acculturation, parental educational expectations, and parent–child relationships. J. Adolesc. Res. 35, 267–294.

[ref53] Lee E. H. Grogan-Kaylor A. Berger L. M. (2022). Parenting practices and child behavior problems in kindergarten: mediating and moderating effects of parent–child relationship quality. Child Youth Serv. Rev. 133:105398

[ref54] Liang L. Bian H. (2022). How to improve the effectiveness of family education guidance services in primary and secondary schools?—based on a study of 113 counties in 9 provinces (autonomous regions and municipalities) in China. J. Chin. Educ. 12, 34–39.

[ref55] Ling F. Li G. C. Zhang J. R. Pi D. D. Lai R. (2018). Parent–child relationships and school bullying among elementary school students: the mediating role of self-sustaining behaviors. Chin. J. Clin. Psychol. 26, 1178–1181. doi: 10.16128/j.cnki.1005-3611.2018.06.027

[ref56] Liu S. Bai Y. Jiang M. Ma D. (2022). A study on the demand for family education guidance service for parents of junior high school students. Tibet Educ. 6, 43–46.

[ref57] Liu G. Ma S. Li J. Zhan W. (2021). Study on the influence of parent–child relationship on children’s behavior development. Surv. Educ. 10, 85–87. doi: 10.16070/J.CNKI.CN45-1388/G4S.2008

[ref58] Liu J. Meng H.-M. (2009). On Bronfenbrenner’s ecosystem theory of developmental psychology. China J. Health Psychol. 17, 250–252. doi: 10.13342/j.cnki.cjhp.2009.02.045

[ref59] Liu X. Wang J. Li Y. Liu X. Li X. (2021). Parental psychological control and educational anxiety among Chinese adolescents: the mediating role of parent–child conflict. Front. Psychol. 12:663726. doi: 10.3389/fpsyg.2021.790923, PMID: 35411208 PMC8993680

[ref61] Luan W. J. Lu H. H. Tong Y. L. Lv D. (2013). The influence of family relationships on the mental health of migrant children. Presch. Educ. Res. 2, 27–36. doi: 10.13861/j.cnki.sece.2013.02.002

[ref62] Mellon R. C. Moutavelis A. G. (2011). Parental educational practices in relation to children’s anxiety disorder-related behavior. J. Anx. Dis. 25, 829–834. doi: 10.1016/j.janxdis.2011.04.003, PMID: 21565462

[ref63] Minuchin S. (2017). Families and family therapy. New York: Routledge.

[ref64] National Association of School Psychologists (2018). Addressing student social, emotional, and behavioral health through a multi-tiered system of support. Available at: https://www.nasponline.org/resources-and-publications/resources/school-safety-and-crisis/multi-tiered-systems-of-support/addressing-student-social,-emotional,-and-behavioral-health-through-multi-tiered-systems-of-support (Accessed June 1, 2023).

[ref66] Ontario Ministry of Education (2018). Policy statement on homeschooling. Available at: https://www.edu.gov.on.ca/extra/eng/ppm/131.html (Accessed June 1, 2023).

[ref67] Qiang Y. (2021). Family education legislation should emphasize the “enhancement of family education capacity”. J. Edu. Sci. Hunan Normal Univ. 3, 55–63. doi: 10.19503/j.cnki.1671-6124.2021.03.007

[ref68] Rey J. M. Schrader E. Morris-Yates A. (1992). Parent-child agreement on children’s behaviours reported by the child behaviour checklist (CBCL). J. Adolesc. 15, 219–230. doi: 10.1016/0140-1971(92)90026-2, PMID: 1447409

[ref69] Robinson K. E. Hoyt L. T. (2021). Parent–child relationship quality and adolescent problem behaviors: the mediating roles of self-esteem and depression. J. Adolesc. 91, 78–89.

[ref70] Scharf M. Mayseless O. (2021). Parental academic involvement, academic anxiety, and children’s social skills: a longitudinal study from early childhood to adolescence. J. Educ. Psychol. 113, 434–449.

[ref71] Schoppe-Sullivan S. J. Braungart-Rieker J. M. Stansbury K. (2020). “Parent–child relationships” in Handbook of life course health development. eds. Forrest C. B. Halfon N. Lerner R. M. (New York: Springer), 295–310.

[ref72] Shang B. (2015). The application of parental authority questionnaire in health field. Wuhan: Wuhan Institute of Physical Education.

[ref73] Shumow L. Vandell D. L. Posner J. K. (2020). Parents’ educational expectations and children’s academic achievement: a cross-cultural perspective. Parent. Sci. Pract. 20, 281–300.

[ref74] Skibbe L. E. Thompson S. F. Downer J. T. Justice L. M. (2021). Parent–child conflict and children’s behavior regulation in the preschool classroom. Early Child Res. Q. 54, 22–32.

[ref75] Strauss G. P. (2021). A bioecosystem theory of negative symptoms in schizophrenia. Front. Psych. 12:655471. doi: 10.3389/fpsyt.2021.655471, PMID: 33841217 PMC8026872

[ref76] Sun J. (2022). A study of the relationship between parenting styles, self-esteem, sense of meaning in life, and academic engagement among high school students. Master’s thesis. [Harbin (Heilongjiang)]: Harbin Normal University.

[ref60] Tudge J. R. Mokrova I. Hatfield B. E. Karnik R. B. (2009). Uses and misuses of Bronfenbrenner’s bioecological theory of human development. J. Fam. Theory Rev. J. 1, 198–210.

[ref77] U.K. Government (1996). Education act 1996. London: The Stationery Office.

[ref78] United Nations (1989). Convention on the rights of the child. New York: United Nations.

[ref79] Walsh F. (2016). Family resilience: a framework for clinical practice. Fam. Process 42, 1–18. doi: 10.1111/j.1545-5300.2003.00001.x 12698595

[ref80] Wang M. Guo J. Chen Q. (2022). Parental academic pressure and children’s behavioral and emotional problems: the mediating roles of parental autonomy support and children’s self-control. J. Child Fam. Stud. 31, 72–84.

[ref81] Wang Y. Zhou H. (2022). The moderating role of authoritative parenting style in the relationship between parental academic expectations and adolescent academic achievement: a meta-analysis. J. Adolesc. 96, 161–170.

[ref82] Wu X. Li M. Wang T. (2022). The relationship between parental academic pressure and students’ academic anxiety in China: a cross-sectional study. Front. Psychol. 13:804.

[ref83] Xia Y. (2016). “Parental responsibility” in the perspective of comparative law. Northern Law J. 1, 25–34. doi: 10.13893/j.cnki.bffx.2016.01.003

[ref84] Xu W. W. (2022). The influence of parent–child games on pupils’ problem behaviors: The mediating role of parent–child relationship [dissertation]. [Guangzhou (Guangdong)]: Sun Yat-sen University.

[ref85] Xu Y. Farver J. A. Zhang Z. Zeng Q. (2019). Parent–child relationships and social-emotional development in Chinese preschoolers: the moderating role of parenting stress. J. Child Fam. Stud. 28, 2760–2772.

[ref86] Yang Y. Xia M. (2018). Reasonable educational anxiety can promote children’s learning and development. Int. J. Pediatr. Adolesc. Med. 5, 41–44.

[ref87] Yang W. Zeng B. (2022). Research on the construction of a professional team of family education guidance services: based on the law of the People’s Republic of China on the promotion of family education. J. Chengdu Norm. Coll. 38, 7–12.

[ref88] Yin X. Liu Y. C. Zhang H. Tu W. T. (2022). Parental expectation bias and educational anxiety. Youth Stud. 1:40-48 + 95.

[ref89] Yin H.-B. Wang K.-T. Zheng X.-P. (2021). Parental emotional support and conflict management in the relationship between parental educational expectations and adolescents’ academic achievement: a moderated mediation model of educational anxiety and parent-adolescent conflict. J. Child Fam. Stud. 30, 814–826. doi: 10.1007/s10826-020-01886-6

[ref90] Yu X. (2010). Study on behavior problems and influencing factors of primary school students [dissertation]. [Wuhan (Hubei)]: Huazhong University of Science and Technology.

[ref91] Yu S. Zheng J. Xu Z. Zhang T. (2022). The transformation of parents’ perception of education involution under the background of “double reduction” policy: the mediating role of education anxiety and perception of education equity. Front. Psychol. 13:800039. doi: 10.3389/fpsyg.2022.800039, PMID: 35664177 PMC9161288

[ref92] Zhang G. (2022). On the order-supporting function of family education: a theoretical investigation from the traditional Chinese family training. J. East China Univ. Pol. Sci. Law. 25, 18–27.

[ref93] Zhong X. Wang Y. Qian M. (2021). Parental academic pressure and children’s depressive symptoms: the mediating role of academic stress and the gender differences. Child Youth Serv. Rev. 120:105842

[ref1001] Zhou Shiyi. (2022). Research on optimization strategy of family education guidance service system in primary and secondary schools in the new era [D]. East China Normal University. doi: 10.27149/d.cnki.ghdsu.2022.002436

[ref94] Zu J. Yang W. Zhou T. Teng W. But F. (2022). The influence of parents’ bowing behavior on children’s problem behavior: a moderating mediation model. Presch. Educ. Res. 6, 34–48. doi: 10.13861/j.cnki.sece.2022.06.013

